# miR-33a is a tumor suppressor microRNA that is decreased in prostate cancer

**DOI:** 10.18632/oncotarget.19521

**Published:** 2017-07-24

**Authors:** Omer Faruk Karatas, Jianghua Wang, Longjiang Shao, Mustafa Ozen, Yiqun Zhang, Chad J. Creighton, Michael Ittmann

**Affiliations:** ^1^ Department of Pathology and Immunology and Michael E. DeBakey Department of Veterans Affairs Medical Center, Houston, TX, USA; ^2^ Department of Molecular Biology and Genetics, Erzurum Technical University, Erzurum, Turkey; ^3^ Department of Medical Genetics, Istanbul University Cerrahpasa Medical School, Istanbul, Turkey; ^4^ Dan L. Duncan Cancer Center Division of Biostatistics, Houston, TX, USA; ^5^ Department of Medicine, Baylor College of Medicine, Houston, TX, USA

**Keywords:** prostate cancer, miR-33a, PIM1, SREBF2, CPT1A

## Abstract

Prostate cancer is one of the most frequently diagnosed neoplasms among men worldwide. MicroRNAs (miRNAs) are involved in numerous important cellular processes including proliferation, differentiation and apoptosis. They have been found to be aberrantly expressed in many types of human cancers. They can act as either tumor suppressors or oncogenes, and changes in their levels are associated with tumor initiation, progression and metastasis. miR-33a is an intronic miRNA embedded within SREBF2 that has been reported to have tumor suppressive properties in some cancers but has not been examined in prostate cancer. SREBF2 increases cholesterol and lipid levels both directly and via miR-33a action. The levels of SREBF2 and miR-33a are correlated in normal tissues by co-transcription from the same gene locus. Paradoxically, SREBF2 has been reported to be increased in prostate cancer, which would be predicted to increase miR-33a levels potentially leading to tumor suppression. We show here that miR-33a has tumor suppressive activities and is decreased in prostate cancer. The decreased miR-33a increases mRNA for the PIM1 oncogene and multiple genes in the lipid β-oxidation pathway. Levels of miR-33a are not correlated with SREBF2 levels, implying posttranscriptional regulation of its expression in prostate cancer.

## INTRODUCTION

Prostate cancer (PCa) is one of the most commonly diagnosed neoplasms and the fifth leading cause of cancer-related death among men in worldwide. More than 650,000 men are diagnosed with PCa annually, and this constitutes almost 10% of all new cancer cases in men worldwide [1]. Up to 30% of PCa patients experience a relapse after a radical prostatectomy [2]. Therefore, a comprehensive characterization of molecular mechanisms associated with prostate carcinogenesis is of paramount importance for enhancing the patient survival and increasing the success of the current therapeutic options [3, 4].

MicroRNAs (miRNAs) are a class of highly conserved and endogenously synthesized gene expression regulators, which are single stranded, non-coding RNA molecules typically 18 to 22 nucleotides long in mature form [5]. MiRNAs specifically interact with their targets predominantly in the post-transcriptional level and inhibit their expression through incorporating into the RNA-induced silencing complex, binding with partial complementarity to their target sites in 3′ untranslated region (3′UTR) of specific mRNAs, and triggering either mRNA degradation or translational repression [6, 7]. MiRNAs are predicted to modulate the expression of at least 60% of human genes [6], and are involved in numerous important cellular processes including proliferation, cell cycle, differentiation, and apoptosis [6, 8, 9]. In recent years, several miRNAs have been found to be aberrantly expressed in distinct types of human cancers including PCa [10, 11]. By targeting cancer associated genes, some miRNAs behave as either tumor suppressors or oncogenes, and changes in their expression are associated with tumor initiation, progression and/or metastasis [4, 12, 13]. A more complete understanding of the roles of unique dysregulated miRNAs in specific cancer types might help unraveling the underlying molecular mechanisms of carcinogenesis and identifying novel putative therapeutic targets, which will enhance the clinical outcome of cancer patients [14].

MiR-33a, is a highly conserved intronic miRNA, is located within the intron 16 of sterol-response-element-binding protein gene, *SREBF2*, on chromosome 22 [15]. There is strong evidence that in normal tissues miR-33a levels are increased by increased transcription of SREBF2, resulting in coordinate regulation of cholesterol and other lipid levels by SREBF2 and miR-33a [15]. SREBF2 has recently been shown to be increased in PCa and to act as an oncogene [16]. However, MiR-33a has been implicated as a tumor suppressor miRNA in a number of malignancies including lung cancer [17–19], breast cancer [20], pancreatic cancer [14], osteosarcoma [21], and melanoma [22] through targeting multiple oncogenic genes [14, 22–24], although miR-33a has not been examined in PCa.

The potential expression of an oncogene and a tumor suppressor from a single genetic locus creates a paradox in PCa. In this study, we show that miR-33a acts as a tumor suppressor in PCa. Furthermore, we show that miR-33a expression is decreased and is not correlated with SREBF2 mRNA levels, implying posttranscriptional mechanisms of control of miR-33a levels in PCa, leading to decreased miR33a levels. The decreased miR-33a levels result in increased levels of mRNAs encoding the PIM1 oncogene and genes promoting β-oxidation of fatty acids.

## RESULTS

### MiR-33a is downregulated in prostate tumor samples and PCa cell lines

**Figure 1:**
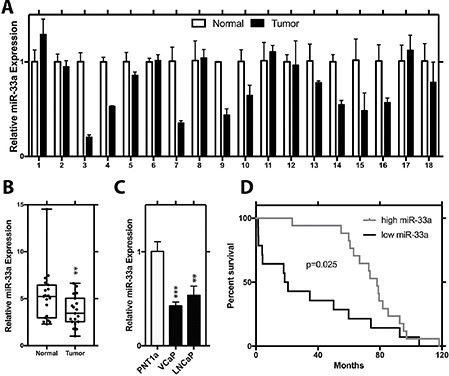
Decreased expression of miR-33a in prostate cancer (**A**) Relative expression of miR-33a in tumor and normal sample pairs. (**B**) Mean relative expression level of miR-33a in tumor tissue samples with respect to normal prostate specimens. (**C**) Endogenous relative expression level of miR-33a in PCa LNCaP and VCaP cell lines and immortalized prostate epithelial cell line (PNT1a). (**D**) Survival analysis for miR-33a in a microarray data set. MiRNA levels were normalized to RNU43, Mean +/− SEM is shown for A-C. *P < 0.05, **P < 0.01, ***P < 0.001; *t*-test.

To explore the biological relevance of miR-33a in PCa, we initially evaluated its relative expression level in 18 pairs of PCa and matched benign tissues from radical prostatectomy specimens. Q-RT-PCR results showed that miR-33a level in more than half of the patients' tumor samples were lower in comparison to adjacent benign prostate samples (Figure 1A). Evaluation of miR-33a expression in all tumor and benign samples demonstrated an average of almost 1.5-fold decrease in cancer tissues (*p* < 0.01, *t*-test; Figure 1B). Comparison of miR-33a levels in PCa cell lines LNCaP, and VCaP cell lines to the immortalized benign prostate epithelial cell line PNT1a showed a significantly lower level of miR-33a level in the PCa cell lines (*p* < 0.05, *t*-test; Figure 1C). We then analyzed a microarray dataset which profiled miRNAs in recurrent vs. non-recurrent PCa samples and demonstrated that reduced miR-33a expression was significantly correlated with poor patient survival (Figure 1D).

### MiR-33a acts as tumor suppressor in PCa

**Figure 2:**
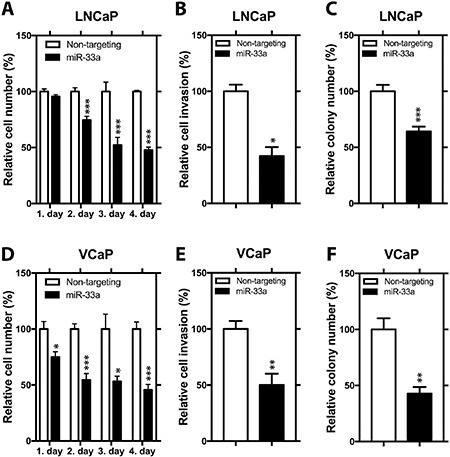
Functional impact of miR-33a overexpression (**A**) Proliferation, (**B**) invasion and (**C**) anchorage independent growth of LNCaP cells transfected with miR-33a mimic. (**D**) Proliferation, (**E**) invasion and (**F**) anchorage independent growth of VCaP cells transfected with miR-33a mimic. Mean +/− SEM is shown *P < 0.05, **P < 0.01, ***P < 0.001; *t*-test.

To analyze the functional impact of miR-33a on PCa cells, we transfected LNCaP and VCaP cells with non-targeting miR or chemically synthesized mature miR-33a, which mimics the endogenous mature miR-33a function and evaluated the alterations in cell proliferation, invasion and soft agar colony formation. We first tested the effectiveness of miR-33a mimic transfection in cells with miRNA Q-RT-PCR. 24 hours after transfection, mature miR-33a levels reached up to ∼15 and ∼20 fold higher in LNCaP and VCaP cells, respectively, compared to corresponding controls (Supplementary Figure 1A, 1C). In functional assays in LNCaP cells, ectopic miR-33a diminished proliferation by up to 53%, invasion by 58% and soft agar colony formation by up to 36% (Figure 2A–2C). In VCaP cells, miR-33a overexpression reduced the potential of proliferation by up to 55%, invasion by 50% and soft agar colony formation by up to 57% (Figure 2D–2F).

**Figure 3:**
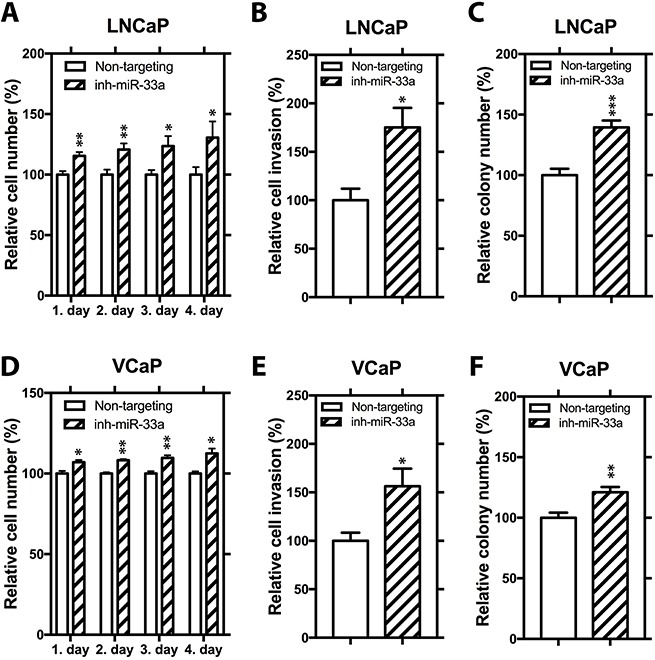
Functional impact of miR-33a inhibition (**A**) Proliferation, (**B**) invasion and (**C**) anchorage independent growth of LNCaP cells transfected with miR-33a inhibitor. (**D**) Proliferation, (**E**) invasion and (**F**) anchorage independent growth of VCaP cells transfected with miR-33a inhibitor. Mean +/− SEM is shown *P < 0.05, **P < 0.01, ***P < 0.001; *t*-test.

We then analyzed the effects of miR-33a inhibition on cellular phenotypes associated with PCa progression by transfecting LNCaP and VCaP cells with non-targeting inhibitor control and synthetic miR-33a inhibitor, which effectively binds to endogenous mature miR-33a and prevent it from functioning, but not degrading it. (Supplementary Figure 1B, 1D). In LNCaP cells, miR-33a suppression increased proliferation by 30%, invasion by 75% and soft agar colony formation by up to 39% (Figure 3A–3C). In VCaP cells, miR-33a inhibition increased proliferation by up to 12% (modest, but statistically significant), invasion by 56% and soft agar colony formation by up to 21% (Figure 3D–3F). Taken together these studies show that miR-33a has biological functions *in vitro* consistent tumor suppression.

### Identification of potential miR-33a targets in PCa

**Figure 4:**
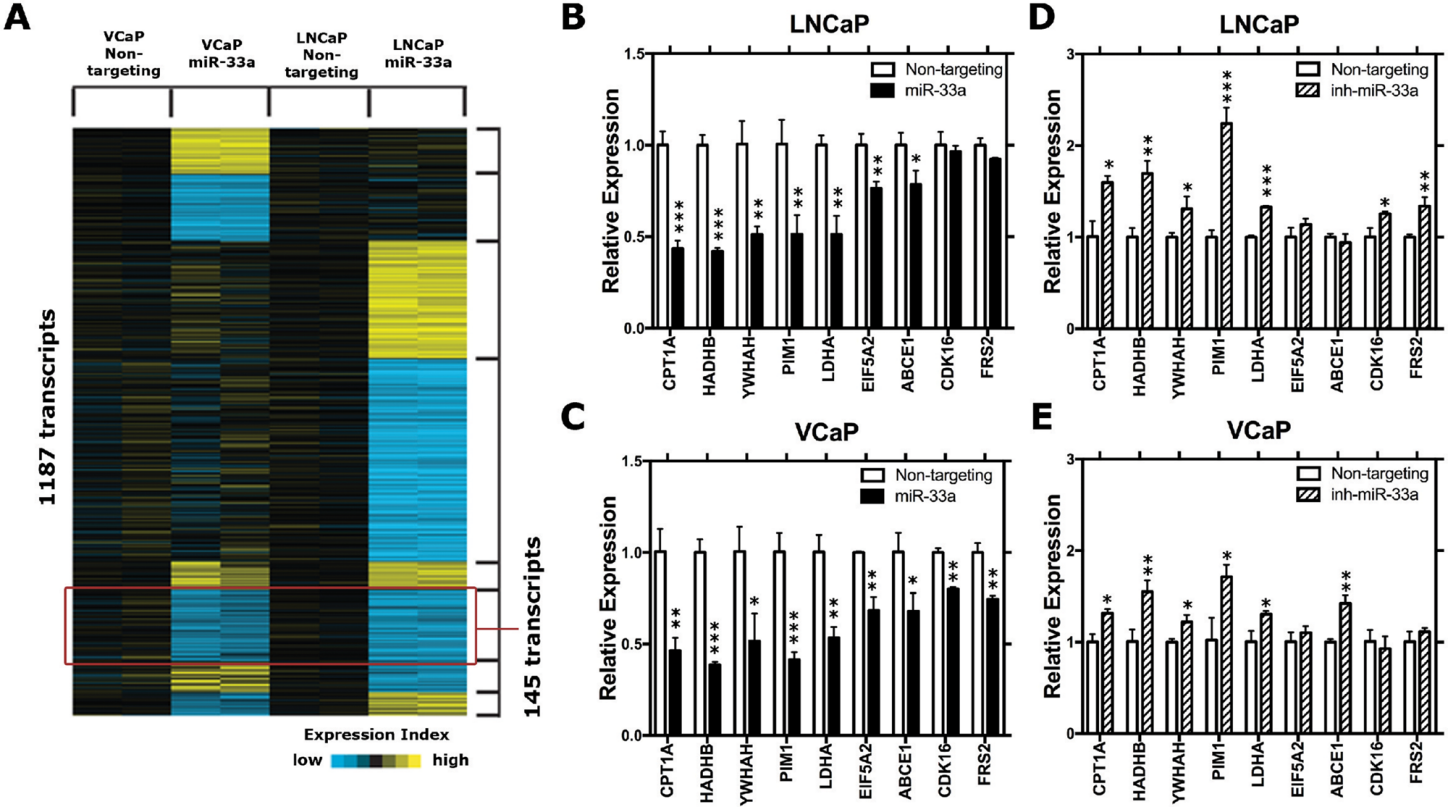
MiR-33a target genes (**A**) The heat-map representation of differentially expressed genes in miR-33a transfected LNCaP and VCaP cells. (**B**) QRT-PCR validation of selected potential miR-33a target genes in miR-33a mimic transfected (B) LNCaP and (**C**) VCaP cells and in miR-33a inhibitor transfected (**D**) LNCaP and (**E**) VCaP cells compared to the control cells. mRNA levels were normalized to β-actin. Mean +/− SEM is shown for B–E; *P < 0.05, **P < 0.01, ***P < 0.001; *t*-test.

To identify putative miR-33a targets in PCa cells, we employed gene expression arrays to identify changes in gene expression upon miR-33a overexpression in both VCaP and LNCaP cells. A total of 1187 probes showed significant up (1.4-fold) or down (0.7-fold) changes in expression. Among those, 145 probes were significantly downregulated in both LNCaP and VCaP cells (Figure 4A). Further *in silico* analysis, to prioritize those potential target genes, demonstrated that 24 candidate genes were predicted to be direct targets of miR-33a by at least 3 miRNA target prediction tools (Supplementary Table 1). We then refined those 24 candidate genes after a comprehensive literature search and selected 9 genes for further Q-RT-PCR validation: CPT1A, HADHB, YWHAH, PIM1, LDHA, EIF5A2, ABCE1, CDK16, and FRS2. Overexpression of miR-33a resulted in significant downregulation of all of these genes except CDK16 and FRS2 in LNCaP cells (Figure 4B), whereas it reduced the expression of all of them in VCaP cells (Figure 4C). In the converse experiment, suppression of miR-33a confirmed CPT1A, HADHB, YWHAH, PIM1, LDHA, ABCE1, CDK16, and FRS2 as potential targets in at least one of the LNCaP and VCaP cells (Figure 4D, 4E). After confirming the gene expression array results with Q-RT-PCR, we selected PIM1 for further analysis, since its dysregulation after changes in miR-33a level was the most significant.

### MiR-33a downregulates PIM1 through directly targeting its 3′UTR of PIM1

**Figure 5:**
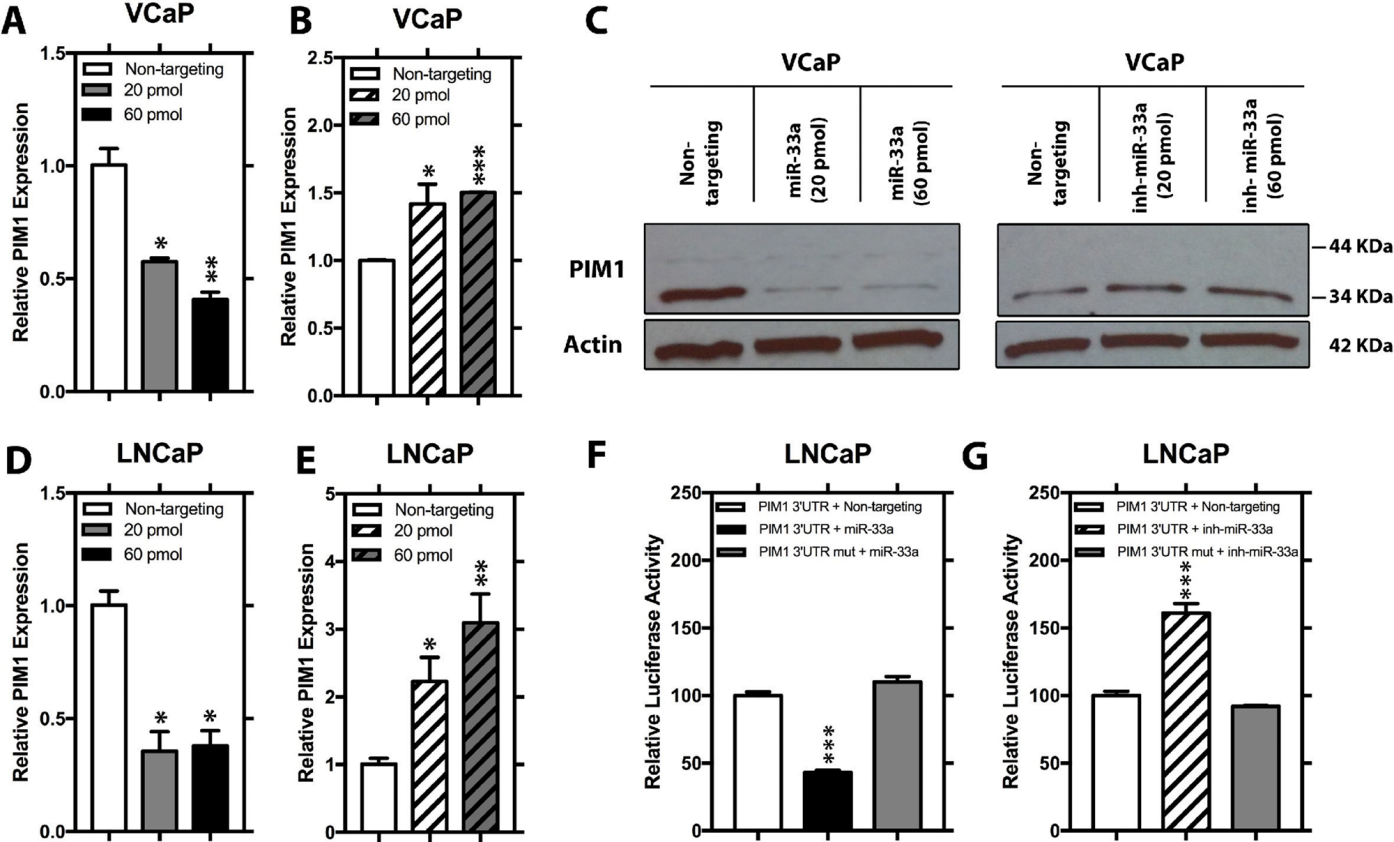
PIM1 expression is repressed by 3′UTR binding of miR-33a Relative mRNA level of PIM1 in VCaP cells transfected with either 20 pmol or 60 pmol (**A**) mimic miR-33a or (**B**) miR-33a inhibitor. (**C**) Relative protein level of PIM1 in VCaP cells transfected with either 20 pmol or 60 pmol mimic miR-33a or miR-33a inhibitor. Relative mRNA level of PIM1 in LNCaP cells transfected with either 20 pmol or 60 pmol (**D**) mimic miR-33a or (**E**) miR-33a inhibitor. Relative luciferase activity of wild-type and mutated PIM1 3′UTR in LNCaP cells transfected with (**F**) mimic miR-33a or (**G**) miR-33a inhibitor. mRNA levels were normalized to β-actin and protein levels were normalized to β-actin, Mean +/− SEM is shown; *P < 0.05, **P < 0.01, ***P < 0.001.

To validate whether miR-33a directly targets PIM1, we initially transfected VCaP cells with 2 different concentrations of mature miR-33a mimic or miR-33a inhibitor and analyzed the expression of PIM mRNA and protein. Q-RT-PCR and western blot analysis demonstrated that higher concentrations of miR-33a mimic lead to larger decreases in PIM1 expression while miR-33a inhibitor resulted in increased PIM1 (Figure 5A–5C).

*In silico* analysis revealed a broadly conserved sequence among vertebrates between positions 734–741 of PIM1 3′UTR as a potential target for mir-33a (Supplementary Figure 2). Transfection of LNCaP with miR-33a or its inhibitor resulted in decreased and increased PIM1 mRNA, respectively Figure 5D–5E. Co-transfecting LNCaP cells with miR-33a mimics or inhibitors together with a luciferase reporter clone containing either the wild-type PIM1 3′UTR or the same region with a mutation in the predicted seed sequence revealed that increased miR-33a repressed the luciferase activity of the wild-type PIM1 3′UTR, however, not that of the PIM1 3′UTR Mut compared to non-targeting control (Figure 5F). Moreover, co-transfection of miR-33a inhibitor resulted in a significant increase in the luciferase activity of the wild-type PIM1 3′UTR, although no change was detected in the mutant reporter construct activity (Figure 5G). Taken together, these results show that PIM1 as a direct target of miR-33a in PCa cell lines.

### MiR-33a reverses the effects of PIM1 on cellular phenotypes associated with PCa progression

**Figure 6:**
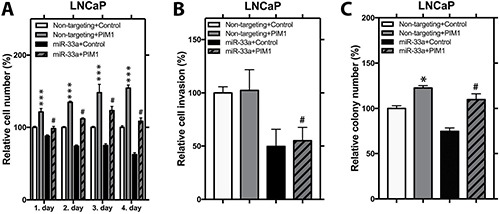
PIM1 expression increases proliferation and anchorage independent growth (**A**) Proliferation, (**B**) invasion and (**C**) anchorage independent growth of control and PIM1 overexpressing LNCaP cells transfected with miR-33a mimic. Mean +/− SEM is shown; *P < 0.05, **P < 0.01, ***P < 0.001; *t*-test. ^#^*P* < 0.05 when non-targeting+PIM1 is compared to miR-33a+PIM1 group.

PIM1, one of the constitutively active kinases, has been reported to act as an oncogene in various human hematological malignancies and solid tumors including PCa [24]. To help understanding the impact of miR-33a on the activity of PIM1 in PCa, we produced LNCaP cells stably overexpressing PIM1 with a wild type 3′UTR. MiR-33a mimic transfection resulted in reduction in PIM1 mRNA and protein levels (Supplementary Figure 3A, 3B). In LNCaP PIM1 cells, ectopic PIM1 expression promoted proliferation by up to 54% and soft agar colony formation by up to 22% (Figure 6A, 6B). Furthermore, introduction of exogenous miR-33a in LNCaP PIM1 cells significantly reduced the induction of PIM1 on proliferation to 8% and soft agar colony formation to 9% (Figure 6A, 6B). Interestingly, in LNCaP PIM1 cells, ectopic PIM1 expression did not alter the invasion (Figure 6C). However, introduction of exogenous miR-33a in both control cells and PIM1 overexpressing cells resulted in similar reduction in invasive potential (Figure 6C). To confirm that the effect of miR-33a on the activity of PIM1 in PCa is a direct effect, we also produced LNCaP cells stably overexpressing PIM1 with mutant 3′UTR (PIM1 Mut). Following MiR-33a mimic transfection, we observed higher PIM1 mRNA levels in PIM1 Mut cells compared to PIM1 cells with wild type 3′UTR (Supplementary Figure 4A). PIM1 Mut had a higher rate of proliferation and soft agar colony formation when treated with exogenous miR-33a compared to PIM1 wild type 3′UTR cells (Supplementary Figure 4B and 4C). However, no difference was seen in invasion between Mut and wild type 3′ UTR cells (Supplementary Figure 4D). These findings indicate that miR-33a decreases proliferation and anchorage independent growth at least in part through directly targeting PIM1, however, effects on invasion are carried out through other targets.

### Lack of correlation of SREBF2 and miR33a in PCa

**Figure 7:**
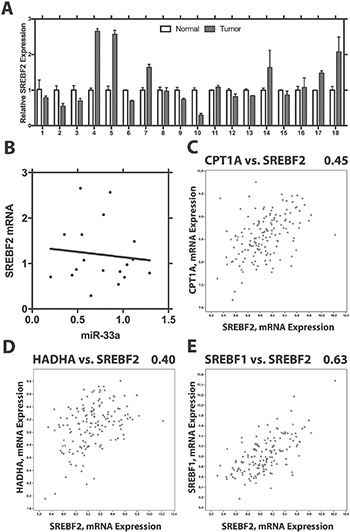
SREBF2 expression is not correlated with levels of miR33a (**A**) Expression of SREBF2 in benign prostate and prostate cancer. Mean +/− SEM of triplicate determinations is shown for 18 pairs. (**B**) Lack of correlation between SREBF2 and miR-33a. Correlation of CPT1A and SREBF2 (**C**) HADHA and SREBF2 (**D**) and SREBF1 and SREBF2 (**E**) in primary PCas in the Taylor dataset using cBioPortal. Pearson r^2^ is shown above each plot.

Having shown that miR-33a is decreased in PCa and acts as a tumor suppressor, we examined the levels of SREBF2 in PCa compared to benign prostate tissue. Using Q-RT-PCR we found variable levels SREBF2 mRNA in PCa, with some cases decreased, some increased and some unchanged (Figure 7A). Overall, the mean level of SREBF2 was increased in PCa, consistent with prior reports [16]. However, there was no correlation of SREBF2 and miR-33a levels in the same cancer samples (Figure 7B; *p* > .6, Pearson). We next examined the Taylor PCa dataset [25] in cBioPortal. Interestingly, we see a strong positive correlation between SREBF2 and the CPT1A, a miR-33a target (Figure 7C), rather than the negative correlation expected if miR-33a was being increased by SREBF2 expression. Similar results were seen with the correlation between SREBF2 and HADHA, which have been shown to be downregulated in parallel to a well-known miR-33a target, HADHB [26] (Figure 7D). Strong positive correlations were seen between SREBF2 and CPT1A, HADHA and HADHB, (a known miR-33A target [26]) in metastatic lesions as well (Supplementary Figure 5A–5C). It has been reported that both SREBF1 and SREBF2 are induced by androgens [27] and are also both induced by decreased miR-185 and miR-342 in PCa [28]. Consistent with this we see a strong correlation of SREBF1 and SREBF2 in primary PCa (Figure 7E) and in metastatic lesion (Supplementary Figure 5D). Overall these results indicate that in PCa SREBF2 expression does not result in increased miR-33a, unlike in many normal tissues and that additional mechanisms lead to decreased miR33a in PCa.

## DISCUSSION

In this report, we show that miR-33a has variable but significantly reduced expression in tumor samples in comparison to their corresponding normal prostate tissues. In addition, decreased miR-33a activity in PCa increases the proliferative, invasive, and anchorage independent growth potential of PCa cells *in vitro* and increased miR33a has the opposite effect. Thus, our data shows that miR-33a is a tumor suppressor in PCa. It should be noted that our *in vitro* experiments used over-expression or silencing which resulted in significant alterations in miR-33a levels. Patient cancer samples showed an average of 1.5-fold decreases in miR-33a. While it is clear that miR-33a is a tumor suppressor, the biological response to this level of loss of miR33a in the human tumors is hard to judge, since the dose response to loss of miR33a in human PCa is not known. The fact that decreased miR-33a in human PCa tumors is associated with more aggressive disease suggests that the losses seen are biologically significant.

MiR-33a, which has been shown to have an important role in the control of lipid and cholesterol metabolism [29], and has been recently implicated as tumor suppressor microRNA in various tumor types [14, 17–22]. To find how miR-33a conducts its functional effects in PCa cells, we examined the potential direct targets of miR-33a in PCa cells via utilizing gene expression microarray analysis and bioinformatics analysis followed by Q-RT-PCR, western blot, and luciferase assay confirmation. Gene expression array and bioinformatics analysis pointed to CPT1A, HADHB, YWHAH, PIM1, LDHA, EIF5A2, ABCE1, CDK16, and FRS2, as direct targets of miR-33a. Since PIM1 is a well-characterized oncogene in PCa [30], we selected it for further confirmation via functional assays, which validated PIM1 as a direct target of miR-33a in PCa cells.

PIM1 has been previously found to have increased expression in high grade prostatic intraepithelial neoplasia [31] and PCa in comparison to normal prostate tissues [32]. PIM1 overexpression has also been found to increase AR transcriptional activity in PCa cell lines, leading to increased cell survival at castrate levels of androgen [33]. Since PIM1 is a constitutively active kinase, its cellular activity is strongly associated with its expression level [24]. While we have not measured PIM1 kinase activity directly in our experiments, the significant alterations in protein levels by mIR33a presumably reflect similar alterations in kinase activity given the impact on cellular phenotypes. PIM1 expression has been reported to be modulated at transcriptional level through activation of JAK/STAT pathway in physiological conditions [34], however, its differential expression in tumor vs. normal cells might be induced through deregulation of microRNAs. Recently, several microRNAs including miR-33a have been found to directly target PIM1 in different cancer types [35, 36]. Our data show strong deregulation of PIM1 expression level upon overexpression or knockdown of miR-33a in PCa cells, which indicates involvement of miR-33a in PCa pathogenesis through altering the oncogenic PIM1 level. Interestingly, our findings show that PIM1 could only alter proliferation and soft agar colony formation abilities, but not invasion of PCa cells. Moreover, miR-33a reverses the impacts of PIM1 on cellular phenotypes associated with PCa progression except cellular invasion. This indicates that the functional effects of miR-33a on cellular invasion may be carried out through other targets. In theory, a single microRNA can target hundreds of mRNAs, therefore, many other targets may also contribute to phenotypes associated with miR-33a deregulation in PCa.

In addition to PIM1, miR-33a has been also shown to directly target a variety of genes associated with cancer progression including several genes associated with cell cycle regulation such as CDK6 (cyclin-dependent kinase 6) and CCND1 (cyclin D1), which are directly targeted by miR-33a [23]. Furthermore, miR-33a has been recently demonstrated to have reduced expression in lung cancer cells and to act as a bone metastasis suppressor in lung cancer through targeting parathyroid hormone related protein [17]. Other miR-33a targets include Twist, HIF-1α and others [17–22, 37]. While these were not among our top hits as miR-33a targets in PCa, they likely contribute to the overall impact of miR-33a on PCa biology as do the targets we identified.

We have also shown that there is no correlation of SREBF2 mRNA with its intronic microRNA miR-33a in PCa unlike the correlation seen in normal tissues. In normal tissues SREBF1 and SREBF2 increase cholesterol and fatty acids by increasing transcription of multiple genes that increase levels of these lipids. MiR-33a targets ABCA1, a cholesterol efflux protein and several mRNAs for proteins involved in β-oxidation of fatty acids including CPT1A and HADHB. Of note, by *in silico* analysis we actually see positive correlation between SREBF2 and CPT1A, HADHA and HADHB in PCa tissues. We also see a positive correlation between SREBF1 and SREBF2 in PCa as expected [38]. Overall, our data indicates that in many PCas, SREBF2 is increased to drive lipid synthesis but via posttranscriptional mechanism, miR-33a is decreased. The decreased miR-33a not only allows upregulation of oncogenic genes such as PIM1 but also allows increased β-oxidation of fatty acids to occur. Such increased β-oxidation may play an important role in providing energy to PCa cells. Consistent with this idea, Schlaepfer et al. [39] have shown that blocking CPT1A activity by knockdown or using drugs leads to decreased growth and apoptosis. Thus increased synthesis and uptake of fatty acids can both increase levels of fatty acids for membrane synthesis but also provide an energy source for PCa, which are known to have low glucose uptake. It is also interesting to note that ABCA1, the cholesterol efflux protein, which is decreased by miR-33a is methylated in PCa [40], which would abrogate the potential deleterious effects of increased cholesterol efflux secondary to decreased miR-33a.

The mechanism by which miR-33a is decreased in PCa is unclear. There are many different mechanisms which have been described [41]. One potential mechanism is via XB130 (also known as AFAP1L2). This actin filament associated protein which is phosphorylated by a number of tyrosine kinases and has been shown to decrease miR-33a [42], although the mechanism is unknown. Of note, XB130 has recently been shown to be increased in PCa and higher expression is associated decreased survival [43]. Further studies will be needed to determine the role of XB130 in miR-33a in PCa.

These studies have potential translational relevance in that metformin, a commonly used drug, decreases miR-33a and that decreased miR-33a has pleiotropic anticancer effects including decreasing c-MYC [44]. A recent meta-analysis concluded that despite significant heterogeneity, metformin was associated with patient benefit in PCa [45]. Our studies suggest that given the variable levels of miR-33a in PCa, those cases with higher miR-33a will most benefit from metformin treatment. Our studies also suggest that PCas with decreased miR-33a, either as a property of the tumor or via metformin, will benefit from inhibition of β-oxidation using drugs as described by Schlaepfer et al. [39]. Further clinical studies will be needed to test these hypotheses.

## MATERIALS AND METHODS

### Benign prostate and prostate cancer specimens

Snap frozen radical prostatectomy specimens were obtained from the Human Tissue Acquisition and Pathology Core of the Dan L. Duncan Cancer Center. Patients were included in the study after obtaining written informed consents under an Institutional Review Board approved protocol. Pathological examination confirmed the presence of at least 70% tumor tissue in cancer samples and the absence of any cancerous tissue in the corresponding benign prostate specimens. RNA was extracted using TRIzol (Invitrogen) method following the manufacturer's protocol.

### Cell culture and miRNA transfection

LNCaP cells were grown in RPMI-1640 medium (GenDepot) supplemented with 10% fetal bovine serum (Gibco) and 100 μg/ml penicillin/ streptomycin (Invitrogen). VCaP cells were cultured in Dulbecco's Modified Eagle Medium (DMEM, Invitrogen) supplemented with 10% fetal bovine serum and 1% penicillin/streptomycin at 37°C in a humidified 5% CO_2_ incubator. Both cell lines were authenticated by STR analysis at MD Anderson Cancer Center Characterized Cell Line Core Facility.

Mature miR-33a mimic and non-targeting mimic control were purchased from Invitrogen. MiR-33a inhibitor and non-targeting inhibitor control were purchased from Sigma. Transfection experiments were optimized and performed using Lipofectamine RNAiMAX Transfection Reagent (Invitrogen) following the manufacturer's protocol.

### cDNA synthesis and quantitative real-time PCR

For miRNA first strand DNA (cDNA) synthesis, miRNA specific primers purchased from Applied Biosystems and “TaqMan MicroRNA reverse transcription Kit (Applied Biosystems) were used to reverse transcribe equal amounts of total RNA. For miRNA expression analysis, TaqMan Fast Advanced Master Mix (Applied Biosystems) was used and microRNA specific probes were purchased from Applied Biosystems. MiRNA expression data were normalized to RNU43.

cDNA synthesis was performed with “amfiRivert cDNA Synthesis Platinum Master Mix” (GenDepot) according to the manufacturers' instructions. For gene expression analysis, SYBR Green PCR Master Mix of Applied Biosystems was used. Expression data were normalized to β-actin. Primer sequences used for Quantitative real time PCR (Q-RT-PCR) are provided in Supplementary Table 2. Q-RT-PCR was carried out in a StepOnePlus™ real-time thermal cycler (Applied Biosystem) using standard parameters. Each experiment was performed in triplicate and the differences in expression levels were evaluated using 2^− ΔΔCT^ method.

### Cell proliferation assay

The proliferative potential of LNCaP and VCaP cells transfected with miR-33a mimic, miR-33a inhibitor or non-targeting miRNA (non-targeting miR) and LNCaP cells stably overexpressing PIM1 in the presence and absence of miR-33a mimic were measured as follows. Cells were plated in 96 well plates at density of 3 × 10^3^ cells per well and after 24 hours they were transfected with miR-33a mimic, miR-33a inhibitor or non-targeting miR using Lipofectamine RNAiMAX Transfection Reagent (Invitrogen). Proliferation rates were measured every 24 hours subsequent to transfection for 4 days. Cell proliferation was evaluated using the Cell Counting Kit-8 (Dojindo) following the manufacturer's instructions by measuring the absorbance at 490 nm with a VERSAmax Tunable microplate reader (Conquer Scientific).

### Matrigel invasion assays

Cell invasion assays were performed using BD BioCoat Matrigel invasion chambers (Becton Dickinson). After transfected with miR-33a mimic, miR-33a inhibitor or non-targeting miR, cells were seeded into invasion chambers at a density of 5 × 10^5^ cells per chamber in triplicates. After 24 hours, non-invading cells were scraped from the upper side of the chambers using a cotton tipped swab and the invading cells on the lower surface of the filter were fixed with 100% methanol and stained with 0.3% crystal violet in 2% ethanol for 20 min. The membranes were then mounted on slides and cells counted.

### Soft agar colony formation assay

Cells transfected with miR-33a mimic, miR-33a inhibitor or non-targeting miR were suspended in a density of 3×10^3^ cells/ml in 0.3% agar diluted in RPMI and plated on a 0.6% base agar in 6-well culture plates. After 14 to 21 days in culture at 37°C, cells were fixed and stained with 0.01% crystal violet in H_2_0 with 10% ethanol and colonies were counted with a dissecting microscope.

### Gene expression array and data analysis

LNCaP and VCaP cells were plated in 6-well plates at a density of 2.5 × 10^5^ cells/well. Two biological replicates of miR-33a or non-targeting miR transfected LNCaP and VCaP cells were collected 24 hours after transfection. Total RNA was extracted using TRIzol reagent (Invitrogen) as described above. Agilent General Human Reference RNA was utilized as reference for array normalization. Total RNAs were labeled, purified, and hybridized to Agilent SurePrint G3 Human Gene Expression 8×60K arrays as described previously [46]. After post-hybridization washes, Agilent Microarray Scanner with Surescan High Resolution Technology was used to scan the slide. Obtained images were analyzed using Feature Extraction v10.7.3.1 (Agilent Technologies, CA, USA) and Bioconductor software (Agilent Technologies). Loess normalization was used to normalize the data. Each miR-33a overexpression profile in VCaP and LNCaP cells was compared to corresponding non-targeting miR controls profiles by fold change using log2 expression values, then top differential genes between overexpression and control groups were selected (with minimum 1.4-fold changes comparing each overexpression profile vs. each control profile) in either cell line. Array data have been deposited into the Gene Expression Omnibus (accession GSE89034).

### *In silico* MicroRNA target prediction and target prioritization

Gene expression array results were refined using miRWalk [47], miRanda [48], RNA22 [49], and TargetScan [50] miRNA target prediction tools to prioritize the putative targets of miR-33a. Common downregulated genes in both LNCaP and VCaP cells upon miR-33a overexpression were ranked according to the number of algorithms that predict the tested gene as a direct target of miR-33a.

### Luciferase reporter assay

A dual luciferase reporter assay was performed to confirm that PIM1 is a direct target of miR-33a in PCa cells. LNCaP cells were seeded in triplicates in 6-well plates at a density of 2.5 × 10^5^ cells/well and co-transfected with PIM1 3′UTR luciferase reporter (Origene, Rockville, MD, USA) and miR-33a mimic, miR-33a inhibitor or non-targeting miR using Lipofectamine 2000 reagent (Thermo Fisher Scientific). Site directed mutagenesis in PIM1 3′UTR luciferase reporter was performed with “QuikChange II XL Site-Directed Mutagenesis Kit” (Agilent) following the manufacturer's instructions. Mutagenesis primer sequences are provided in Supplementary Table 1. Cells were harvested 2 days after transfection and lysed in passive lysis buffer. Lysates were assayed using the Dual Luciferase Assay System (Promega) to quantify relative luciferase activities.

### Stable overexpression of PIM1

PIM1 overexpression vector was generated by cloning of full-length open reading frame and 3′UTR of PIM1 into pcDNA™3.1/V5-His TOPO^®^ vector, containing a neomycin selectable marker, with “pcDNA™3.1/V5-His TOPO^®^ TA Expression Kit” following the manufacturer's instructions. Primer sequences used for cloning are provided in Supplementary Table 2.

LNCaP cells were seeded at a density of 2.5 × 10^5^ cells/well in 6-well plates. After 24 hours, cells were transfected with 2,5 μg of PIM1 overexpression plasmid or control vector with Lipofectamine 3000 (Thermo) following the manufacturer's protocol. Two days after transfection, stable clones were selected with G418 (GIBCO) and then expanded in RPMI growth medium supplemented with G148 for further *in vitro* analysis.

### Western blot analysis

Cells were seeded in duplicates into 6 well plates and transfected with miR-33a mimic, miR-33a inhibitor or non-targeting miR. 48 hours after transfection, cells were scraped from the plates and washed in cold phosphate buffered saline twice. Then cells were lysed in RIPA lysis buffer containing 10 mM PMSF, and 1x protease inhibitors (Pierce) and clarified by centrifugation. Protein concentration was measured using BCA protein assay kit (Thermo Scientific). Equal amounts of protein extracts (20 μg) were subjected to electrophoresis in 10% SDS-PAGE, transferred onto nitrocellulose membrane using iBlot 2 Dry Blotting System (Thermo). Membranes were probed with the primary antibodies diluted in 1x PBST (0.1% Tween-20), 5% skim milk: rb-α-PIM1 (1:2000, Cell Signaling) and m-α-β-Actin (1:5000 [46]. β-Actin was used as loading control. Corresponding HRP-conjugated secondary antibodies (1:3000, Thermo, Rockford USA) were used along with Pierce ECL western blotting substrate (Thermo, Rockford USA) to visualize the signals.

### Analysis of cBioPortal data

Co-expression of SREBF1, SREBF2, CPT1A, HADHA and HADHB was analyzed in the dataset of Taylor et al. [25] in primary and metastatic tumors with mRNA using cBioPortal [51, 52].

### Statistical analysis

Data were plotted as mean ± standard error and the statistical significances were evaluated using Student's *t* test. A *p* value of 0.05 or below was considered as significant. Kaplan-Meier analysis of time to biochemical recurrence of an independent set of PCas was carried out using microarray analysis of miRNA levels (manuscript submitted. M Ozen; see Gene Expression Omnibus accession GSE88958).

## SUPPLEMENTARY FIGURES AND TABLES





## Abbreviations

PCa: prostate cancer
UTR: untranslated region

## References

[1] Quinn M, Babb P (2002). Patterns and trends in prostate cancer incidence, survival, prevalence and mortality. Part I: international comparisons. BJU Int.

[2] Han M, Partin AW, Zahurak M, Piantadosi S, Epstein JI, Walsh PC (2003). Biochemical (prostate specific antigen) recurrence probability following radical prostatectomy for clinically localized prostate cancer. J Urol.

[3] Guzel E, Karatas OF, Duz MB, Solak M, Ittmann M, Ozen M (2014). Differential expression of stem cell markers and ABCG2 in recurrent prostate cancer. Prostate.

[4] Karatas OF, Guzel E, Suer I, Ekici ID, Caskurlu T, Creighton CJ, Ittmann M, Ozen M (2014). miR-1 and miR-133b Are Differentially Expressed in Patients with Recurrent Prostate Cancer. Plos One.

[5] Bushati N, Cohen SM (2007). microRNA functions. Annu Rev Cell Dev Biol.

[6] Bartel DP (2009). MicroRNAs: target recognition and regulatory functions. Cell.

[7] Filipowicz W, Bhattacharyya SN, Sonenberg N (2008). Mechanisms of post-transcriptional regulation by microRNAs: are the answers in sight?. Nat Rev Genet.

[8] Bentwich I, Avniel A, Karov Y, Aharonov R, Gilad S, Barad O, Barzilai A, Einat P, Einav U, Meiri E, Sharon E, Spector Y, Bentwich Z (2005). Identification of hundreds of conserved and nonconserved human microRNAs. Nat Genet.

[9] Ventura A, Jacks T (2009). MicroRNAs and cancer: short RNAs go a long way. Cell.

[10] Seven M, Karatas OF, Duz MB, Ozen M (2014). The role of miRNAs in cancer: from pathogenesis to therapeutic implications. Future Oncol.

[11] Bonci D, De Maria R (2015). A predictive signature for therapy assignment and risk assessment in prostate cancer. Oncoscience.

[12] Ozen M, Karatas OF, Gulluoglu S, Bayrak OF, Sevli S, Guzel E, Ekici ID, Caskurlu T, Solak M, Creighton CJ, Ittmann M (2015). Overexpression of miR-145-5p Inhibits Proliferation of Prostate Cancer Cells and Reduces SOX2 Expression. Cancer Invest.

[13] Karatas OF, Yuceturk B, Suer I, Yilmaz M, Cansiz H, Solak M, Ittmann M, Ozen M (2016). Role of miR-145 in human laryngeal squamous cell carcinoma. Head Neck.

[14] Liang C, Yu XJ, Guo XZ, Sun MH, Wang Z, Song Y, Ni QX, Li HY, Mukaida N, Li YY (2015). MicroRNA-33a-mediated downregulation of Pim-3 kinase expression renders human pancreatic cancer cells sensitivity to gemcitabine. Oncotarget.

[15] Najafi-Shoushtari SH, Kristo F, Li Y, Shioda T, Cohen DE, Gerszten RE, Naar AM (2010). MicroRNA-33 and the SREBP host genes cooperate to control cholesterol homeostasis. Science.

[16] Li X, Wu JB, Li Q, Shigemura K, Chung LW, Huang WC (2016). SREBP-2 promotes stem cell-like properties and metastasis by transcriptional activation of c-Myc in prostate cancer. Oncotarget.

[17] Kuo PL, Liao SH, Hung JY, Huang MS, Hsu YL (2013). MicroRNA-33a functions as a bone metastasis suppressor in lung cancer by targeting parathyroid hormone related protein. Biochim Biophys Acta.

[18] Kang J, Kim W, Lee S, Kwon D, Chun J, Son B, Kim E, Lee JM, Youn H, Youn B (2017). TFAP2C promotes lung tumorigenesis and aggressiveness through miR-183- and miR-33a-mediated cell cycle regulation. Oncogene.

[19] Yang L, Yang J, Li J, Shen X, Le Y, Zhou C, Wang S, Zhang S, Xu D, Gong Z (2015). MircoRNA-33a inhibits epithelial-to-mesenchymal transition and metastasis and could be a prognostic marker in non-small cell lung cancer. Sci Rep.

[20] Zhang C, Zhang Y, Ding W, Lin Y, Huang Z, Luo Q (2015). MiR-33a suppresses breast cancer cell proliferation and metastasis by targeting ADAM9 and ROS1. Protein Cell.

[21] Zhang J, Wang D, Xiong J, Chen L, Huang J (2015). MicroRNA-33a-5p suppresses growth of osteosarcoma cells and is downregulated in human osteosarcoma. Oncol Lett.

[22] Zhou J, Xu D, Xie H, Tang J, Liu R, Li J, Wang S, Chen X, Su J, Zhou X, Xia K, He Q, Chen J, Xiong W, Cao P, Cao K (2015). miR-33a functions as a tumor suppressor in melanoma by targeting HIF-1α. Cancer Biol Ther.

[23] Cirera-Salinas D, Pauta M, Allen RM, Salerno AG, Ramírez CM, Chamorro-Jorganes A, Wanschel AC, Lasuncion MA, Morales-Ruiz M, Suarez Y, Baldan Á, Esplugues E, Fernández-Hernando C (2012). Mir-33 regulates cell proliferation and cell cycle progression. Cell Cycle.

[24] Thomas M, Lange-Grunweller K, Weirauch U, Gutsch D, Aigner A, Grunweller A, Hartmann RK (2012). The proto-oncogene Pim-1 is a target of miR-33a. Oncogene.

[25] Taylor BS, Schultz N, Hieronymus H, Gopalan A, Xiao Y, Carver BS, Arora VK, Kaushik P, Cerami E, Reva B, Antipin Y, Mitsiades N, Landers T (2010). Integrative genomic profiling of human prostate cancer. Cancer Cell.

[26] Gerin I, Clerbaux LA, Haumont O, Lanthier N, Das AK, Burant CF, Leclercq IA, MacDougald OA, Bommer GT (2010). Expression of miR-33 from an SREBP2 intron inhibits cholesterol export and fatty acid oxidation. J Biol Chem.

[27] Swinnen JV, Ulrix W, Heyns W, Verhoeven G (1997). Coordinate regulation of lipogenic gene expression by androgens: evidence for a cascade mechanism involving sterol regulatory element binding proteins. Proc Natl Acad Sci USA.

[28] Li X, Chen YT, Josson S, Mukhopadhyay NK, Kim J, Freeman MR, Huang WC (2013). MicroRNA-185 and 342 inhibit tumorigenicity and induce apoptosis through blockade of the SREBP metabolic pathway in prostate cancer cells. PLoS One.

[29] Ono K (2016). Functions of microRNA-33a/b and microRNA therapeutics. J Cardiol.

[30] Santio NM, Salmela M, Arola H, Eerola SK, Heino J, Rainio EM, Koskinen PJ (2016). The PIM1 kinase promotes prostate cancer cell migration and adhesion via multiple signalling pathways. Exp Cell Res.

[31] Valdman A, Fang X, Pang ST, Ekman P, Egevad L (2004). Pim-1 expression in prostatic intraepithelial neoplasia and human prostate cancer. Prostate.

[32] He HC, Bi XC, Zheng ZW, Dai QS, Han ZD, Liang YX, Ye YK, Zeng GH, Zhu G, Zhong WD (2009). Real-time quantitative RT-PCR assessment of PIM-1 and hK2 mRNA expression in benign prostate hyperplasia and prostate cancer. Med Oncol.

[33] van der Poel HG, Zevenhoven J, Bergman AM (2010). Pim1 regulates androgen-dependent survival signaling in prostate cancer cells. Urol Int.

[34] Bachmann M, Moroy T (2005). The serine/threonine kinase Pim-1. Int J Biochem Cell Biol.

[35] Wang Y, Zhou X, Shan B, Han J, Wang F, Fan X, Lv Y, Chang L, Liu W (2015). Downregulation of microRNA-33a promotes cyclin-dependent kinase 6, cyclin D1 and PIM1 expression and gastric cancer cell proliferation. Mol Med Rep.

[36] Rang Z, Yang G, Wang YW, Cui F (2016). miR-542-3p suppresses invasion and metastasis by targeting the proto-oncogene serine/threonine protein kinase, PIM1, in melanoma. Biochem Biophys Res Commun.

[37] Zhang M, Gong W, Zuo B, Chu B, Tang Z, Zhang Y, Yang Y, Zhou D, Weng M, Qin Y, Ma M, Jiang A, Ma F, Quan Z (2016). The microRNA miR-33a suppresses IL-6-induced tumor progression by binding Twist in gallbladder cancer. Oncotarget.

[38] Swinnen JV, Ulrix W, Heyns W, Verhoeven G (1997). Coordinate regulation of lipogenic gene expression by androgens: evidence for a cascade mechanism involving sterol regulatory element binding proteins. Proc Natl Acad Sci USA.

[39] Schlaepfer IR, Rider L, Rodrigues LU, Gijon MA, Pac CT, Romero L, Cimic A, Sirintrapun SJ, Glode LM, Eckel RH, Cramer SD (2014). Lipid catabolism via CPT1 as a therapeutic target for prostate cancer. Mol Cancer Ther.

[40] Lee BH, Taylor MG, Robinet P, Smith JD, Schweitzer J, Sehayek E, Falzarano SM, Magi-Galluzzi C, Klein EA, Ting AH (2013). Dysregulation of cholesterol homeostasis in human prostate cancer through loss of ABCA1. Cancer Res.

[41] Siomi H, Siomi MC (2010). Posttranscriptional regulation of microRNA biogenesis in animals. Mol Cell.

[42] Takeshita H, Shiozaki A, Bai XH, Iitaka D, Kim H, Yang BB, Keshavjee S, Liu M (2013). XB130, a new adaptor protein, regulates expression of tumor suppressive microRNAs in cancer cells. PLoS One.

[43] Chen B, Liao M, Wei Q, Liu F, Zeng Q, Wang W, Liu J, Hou J, Yu X (2016). XB130 is overexpressed in prostate cancer and involved in cell growth and invasion. Oncotarget.

[44] Blandino G, Valerio M, Cioce M, Mori F, Casadei L, Pulito C, Sacconi A, Biagioni F, Cortese G, Galanti S, Manetti C, Citro G, Muti P, Strano S (2012). Metformin elicits anticancer effects through the sequential modulation of DICER and c-MYC. Nat Commun.

[45] Coyle C, Cafferty FH, Vale C, Langley RE (2016). Metformin as an adjuvant treatment for cancer: a systematic review and meta-analysis. Ann Oncol.

[46] Feng S, Wang J, Zhang Y, Creighton CJ, Ittmann M (2015). FGF23 promotes prostate cancer progression. Oncotarget.

[47] Dweep H, Sticht C, Pandey P, Gretz N (2011). miRWalk--database: prediction of possible miRNA binding sites by “walking” the genes of three genomes. J Biomed Inform.

[48] John B, Enright AJ, Aravin A, Tuschl T, Sander C, Marks DS (2004). Human MicroRNA targets. PLoS Biol.

[49] Miranda KC, Huynh T, Tay Y, Ang YS, Tam WL, Thomson AM, Lim B, Rigoutsos I (2006). A pattern-based method for the identification of MicroRNA binding sites and their corresponding heteroduplexes. Cell.

[50] Garcia DM, Baek D, Shin C, Bell GW, Grimson A, Bartel DP (2011). Weak seed-pairing stability and high target-site abundance decrease the proficiency of lsy-6 and other microRNAs. Nat Struct Mol Biol.

[51] Gao J, Aksoy BA, Dogrusoz U, Dresdner G, Gross B, Sumer SO, Sun Y, Jacobsen A, Sinha R, Larsson E, Cerami E, Sander C, Schultz N (2013). Integrative analysis of complex cancer genomics and clinical profiles using the cBioPortal. Sci Signal.

[52] Cerami E, Gao J, Dogrusoz U, Gross BE, Sumer SO, Aksoy BA, Jacobsen A, Byrne CJ, Heuer ML, Larsson E, Antipin Y, Reva B, Goldberg AP, Sander C, Schultz N (2012). The cBio cancer genomics portal: an open platform for exploring multidimensional cancer genomics data. Cancer Discov.

