# Supplementary material for: miR-33a is a tumor suppressor microRNA that is decreased in prostate cancer
Source: Oncotarget. 2017 Jul 24;8(36):60243–56. doi: 10.18632/oncotarget.19521 (PMC5601135; doi:10.18632/oncotarget.19521)
Supplement: Supplementary file 1 [file oncotarget-08-60243-s001.pdf]

miR-33a is a tumor suppressor microRNA that is decreased in prostate cancer

SUPPLEMENTARY MATERIALS

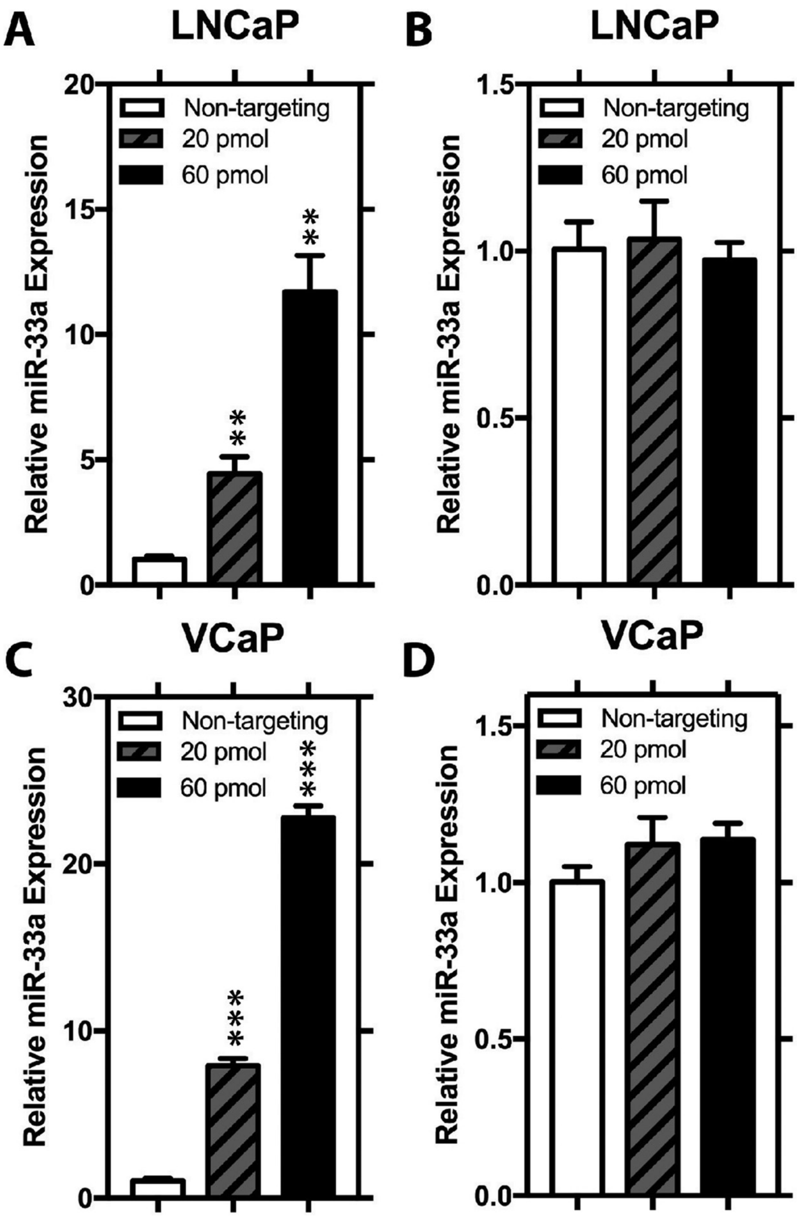

Supplementary Figure 1: MiR-33a level in prostate cancer cells after overexpression or knockdown of miR-33a. Relative expression of miR-33a in LNCaP cells transfected with mimic miR-33a (**A**) or miR-33a inhibitor (**B**). Relative expression of miR-33a in LNCaP cells transfected with mimic miR-33a (**C**) or miR-33a inhibitor (**D**). MiRNA levels were normalized to RNU43, $*P < 0.05$, $**P < 0.01$, $***P < 0.001$.

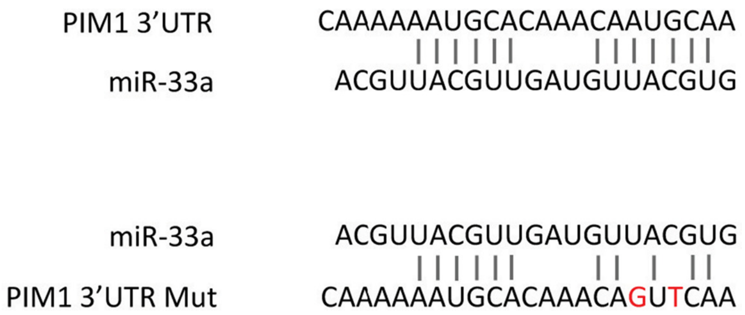

Supplementary Figure 2: MiR-33a target nucleotide sequence on PIM1 3′ UTR. Predicted miR-33a target nucleotide sequence on PIM1 3′ UTR and mutated base pairs as a result of site directed mutagenesis.

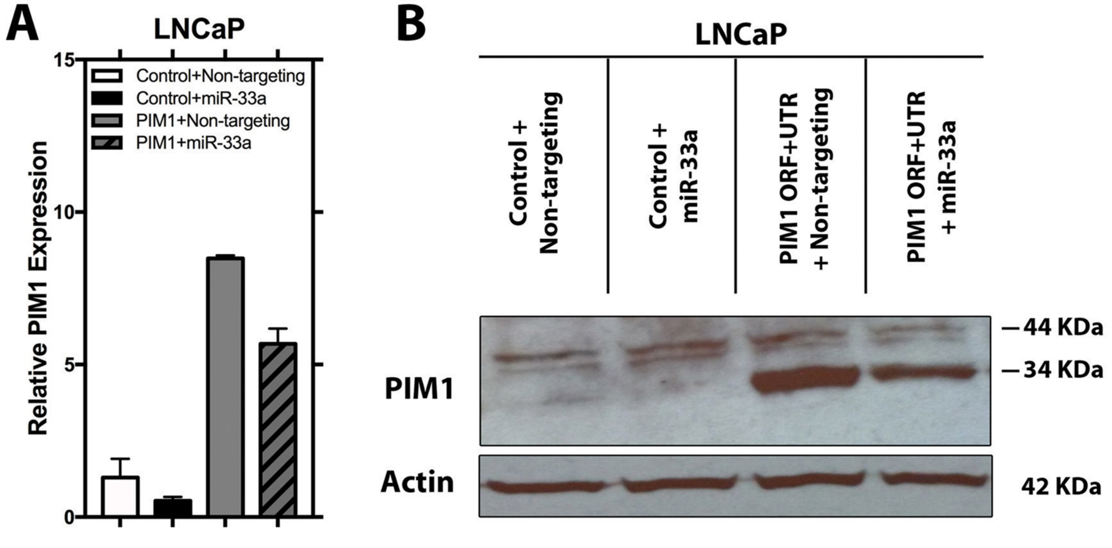

Supplementary Figure 3: PIM1 level in mRNA and protein level in miR-33a mimic transfected control and PIM1 overexpressing LNCaP cells. Relative mRNA (**A**) and protein (**B**) level of PIM1 in control and PIM1 overexpressing LNCaP cells transfected with mimic miR-33a. Gene expression levels were normalized to β-actin, $^{*}P < 0.05$, $^{**}P < 0.01$, $^{***}P < 0.001$.

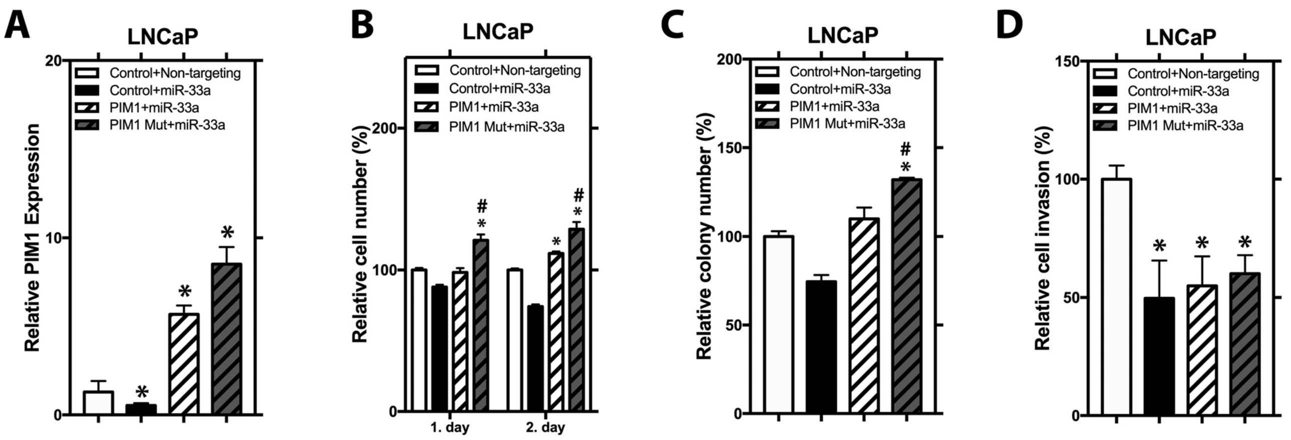

Supplementary Figure 4: PIM1 3′ UTR sequence controls PIM1 expression and phenotype mediated by miR-33a. Relative PIM1 mRNA levels in LNCaP cells stably overexpressing PIM1 with mutant 3′UTR (PIM1 Mut) and controls following MiR-33a mimic transfection (**A**) $^*P < 0.05$ versus control without PIM1. Proliferation (**B**), soft agar colony formation (**C**) and invasion (**D**) were measured in PIM1 Mut and controls treated with exogenous miR-33a. $^*P < 0.05$ versus control without PIM1. $^{\#}P < .05$ PIM1 Mut versus PIM1 wild type 3-UTR.

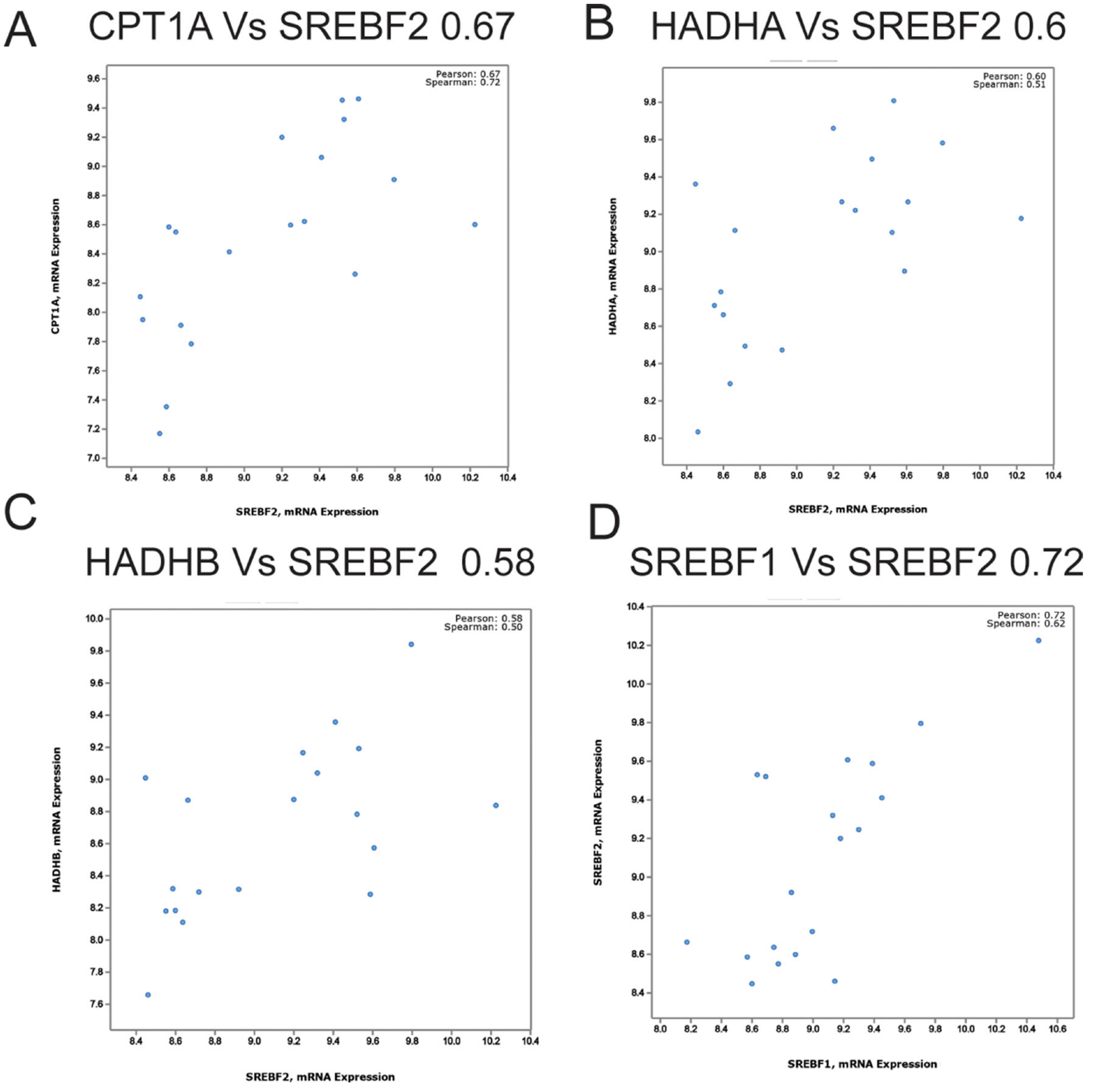

Supplementary Figure 5: Correlation of SREBF2 mRNA and mRNAs of genes associated lipid oxidation and synthesis in metastatic prostate cancer. Correlation of (**A**) CPT1A vs. SREBF2, (**B**) HADHA vs. SREBF2, (**C**) HADHB vs. SREBF2, and (**D**) SREBF1 vs. SREBF2 mRNAs in metastatic PCas in the Taylor dataset using cBioPortal. Pearson r^2 is shown above each plot.

Supplementary Table 1: Predicted binding of MiR-33a to the 3′UTR of genes downregulated by MiR-33a in VCaP and LNCaP PCa cells using four different miRNA target prediction tools. See Supplementary_Table_1

Supplementary Table 2: QRT-PCR primer sequences and PIM1 3′UTR cloning and mutagenesis primer sequences

Gene	Sequence
**Beta-actin-F**	5′-GCCTCGCCTTTGCCGATC-3′
**Beta-actin-R**	5′-CCCACGATGGAGGGGAAG-3′
**PIM1-F**	5′- CCGTCTACACGGACTTCGAT-3′
**PIM1-R**	5′- CTGGCCCCTGATGATCTCTT-3′
**CPT1A-F**	5′- GGCAAGTTTTGCCTCACATACG-3′
**CPT1A-R**	5′- GAACAACTTCAGCCTCTGTTCC-3′
**HADHB-F**	5′- AAACCAAGGTTGGATTGCCTC-3′
**HADHB-R**	5′- CACTATCATAGCATGGCCCTG-3′
**YWHAH-F**	5′- GGTGACAGAGCTGAATGAACC-3′
**YWHAH-R**	5′- TCATTGCAAACTGTCTCCAGC-3′
**ABDE1-F**	5′- GGTTACACCCCAGAGCAAAA-3′
**ABCE1-R**	5′- GGGATAGGCAACCTGTGAAG-3′
**EIF5A2-F**	5′- CACCATGGCAGACGAAATTGA-3′
**EIF5A2-R**	5′- CCAACAAGGTGAACCTTGGC-3′
**LDHA-F**	5′- GTTGCTGGTGTCTCTCTGAA-3′
**LDHA-R**	5′- TCCAATAGCCCAGGATGTGT-3′
**CDK16-F**	5′- AGGACATCAACAAGCGCCTA-3′
**CDK16-R**	5′- GTTTCCCAAAGCCAATCTCAG-3′
**FRS2-F**	5′- GAAGAAGCCATGGGTAGCTG-3′
**FRS2-R**	5′- TCACGTTTGCGGGTGTATAA-3′
**PIM1 ORF+UTR-F**	5′- CCTCAGTTGTCCTCCGACTC-3′
**PIM1 ORF+UTR-R**	5′- GAGAGGATCTGGAAGGCACA-3′
**Mutagenesis-F**	5′ACATTTACAGCTTTTCTGTTGATTGAACTGTTTGTGCATTTTTTGTGTGTGTG-3′
**Mutagenesis-R**	5′CACACACACAAAAAATGCACAAACAGTTCAATCAACAGAAAAGCTGTAAATGT-3′
